# Prevalence of Nocturnal Enuresis and Its Associated Factors in Primary School and Preschool Children of Khorramabad in 2013

**DOI:** 10.1155/2014/120686

**Published:** 2014-10-12

**Authors:** Katayoun Bakhtiar, Yadollah Pournia, Farzad Ebrahimzadeh, Ali Farhadi, Fathollah Shafizadeh, Reza Hosseinabadi

**Affiliations:** ^1^Department of Public Health, Faculty of Health and Nutrition, Lorestan University of Medical Sciences, Khorramabad 6813833946, Iran; ^2^Faculty of Medicine, Lorestan University of Medical Sciences, Khorramabad 6813833946, Iran; ^3^Department of Social Medicine, Lorestan University of Medical Sciences, Khorramabad 6813833946, Iran; ^4^Department of Pediatrics, Lorestan University of Medical Sciences, Khorramabad 6813833946, Iran; ^5^Social Determinants of Health Research Center, Lorestan University of Medical Sciences, Khorramabad 6813833946, Iran

## Abstract

*Background*. Nocturnal enuresis refers to an inability to control urination during sleep. This study aimed to determine the prevalence of nocturnal enuresis and its associated factors in children in the city of Khorramabad. *Materials and Methods*. In this descriptive-analytic, cross-sectional study, 710 male and female children were divided into two groups with equal numbers. The samples were selected from the schools of Khorramabad using the multistage cluster and stratified random sampling methods based on the diagnostic criteria of DSM-IV. The data was analyzed using the logistic regression. *Results*. The results showed that 8% of the children had nocturnal enuresis, including 5.2% of primary nocturnal enuresis and 2.8% of secondary nocturnal enuresis. The prevalence of nocturnal enuresis in the boys (10.7%) was higher compared with that in the girls (5.4%) (*P* = 0.009). There were statistically significant relationships between nocturnal enuresis and history of nocturnal enuresis in siblings (*P* = 0.023), respiratory infections (*P* = 0.036), deep sleep (*P* = 0.007), corporal punishment at school (*P* = 0.036), anal itching (*P* = 0.043), and history of seizures (*P* = 0.043). *Conclusion*. This study showed that the prevalence of nocturnal enuresis in the boys was higher compared with that in the girls.

## 1. Introduction

Nocturnal enuresis refers to an inability to control urination and involuntary urination during sleep, which is common among young children [1]. Based on the DSM-IV (diagnostic and statistical manual of mental disorders IV) criteria, enuresis refers to the urination of children over 5 years old in clothes or in bed that happens twice a week for three consecutive months can occur at night, during the day, or a combination of these two, and is also called nocturnal enuresis [2]. Enuresis is classified as primary enuresis (urinary incontinence in a child who has never been dry) and secondary enuresis (urinary incontinence in a child who has been dry for at least 6 months) [3]. Nocturnal enuresis in children is the second most common disorder after allergic diseases [1]. Nocturnal enuresis can cause a variety of behavioral, psychological, and social problems including embarrassment, blushing, lack of self-esteem, and aggression. Therefore, identifying children at risk and performing therapeutic measures are necessary [1, 3]. Based on the results of various investigations, enuresis has many causes including developmental differences, for example, differences in the growth of the urinary sphincters of a child, various diseases like diabetes, urinary tract infections, and so forth, emotional changes and conflicts such as the birth of a new baby and scholastic or educational stressful conditions, and emotional crises such as parental separation and divorce, family conflicts, and so forth [3–6].

Primary enuresis is often associated with a familial history of delayed urinary bladder control. Secondary enuresis may also be due to urologic and neurological problems, disorders of the spinal cord, and recurrent urinary tract infection [6]. Ninety percent of enuresis cases are primary, and its prevalence usually changes with age [6]. Several studies have been conducted on nocturnal enuresis and its associated factors. The review of the relevant literature showed considerably different prevalence rates for enuresis [3, 4], so that the prevalence rates in different regions and in children older than 5 years old have been reported to be 5–20% [1].

In a study by Unalacak et al. conducted in Turkey, the prevalence of nocturnal enuresis was 8.9%, out of which 7.75% was primary nocturnal enuresis [7]. Also, studies indicated the prevalence rates of 4.7% in China [8] and 18.2% in Australia in which 12.3%, 2.5%, and 3.6% of the children were reported to have mild, moderate, and severe nocturnal enuresis, respectively [9]. In the US, 5–10% of 7-year-old children suffered from nocturnal enuresis while the rate was lower than 5% in children over 10 years old [10]. The prevalence rate of nocturnal enuresis in Ethiopia has been reported to be 5% in small towns, 0.8% in most rural areas, and 9% in big cities [11].

Considering the importance and consequences of nocturnal enuresis and lack of a comprehensive study in this regard in the city of Khorramabad (west of Iran), this study aimed to investigate the prevalence of this disorder and its associated factors in 5–10-year-old children.

## 2. Materials and Methods

The city of Khorramabad (west of Iran) has two educational areas and 35 public and private schools. Out of this number, 24 schools were selected from the northern, southern, and central areas through the systematic random sampling, so that the distribution of the schools on the map of the city was homogeneous. The method used to select the samples was a combination of the stratified random sampling and the multistage cluster sampling. The educational levels (elementary school and preschool) were considered as two separate strata, and in each stratum, the northern, central, and southern areas of the city were in turn considered as three substrata. In each substratum, different schools were considered as cluster heads and a total of 24 schools were selected via the systematic random sampling from among the schools (cluster heads). Then, in each school, any educational level (grade) was considered as a stratum, one class was selected from each educational level, and the samples were selected from the class through the systematic randomization. The minimum sample size needed to investigate the prevalence of enuresis was calculated to be 708 samples (354 girls and 354 boys), considering similar studies, the significance level of 0.05, and the precision of 0.02. After the samples were selected, their parents attended the schools in coordination with the school principal, completed written informed consents, and completed the questionnaire with the help of the interviewer. The tool applied in this study was a research-made questionnaire that was prepared based on reviewing the relevant literature and evaluating factors associated with enuresis. The content validation was applied to examine the validity of the questionnaire. To do this, the expert viewpoints of six specialists were considered, including 2 pediatricians, 2 biostatisticians, 1 health education specialist, and 1 child psychologist. To determine the reliability of the questionnaire, the test-retest reliability was applied on a sample size of 30 people with an interval of one week. The reliability was confirmed with a correlation coefficient of more than 0.8 and the significance level of 0.05. Additionally, Kappa's agreement coefficient of 0.8 was applied for the nominal scales for this purpose.

Four interviewers were trained in collecting the data and were calibrated with the researcher with a coefficient of 75%. The questionnaire applied in the study consisted of two parts. The first part contained the demographic and socioeconomic data including age, gender, parental marital status, parental education, parental kinship, parental occupation, family size, birth order, and other variables such as new baby's birth in the family, change of living place, sleep quality, child's corporal punishment by parents, academic failure, and corporal punishment at school. The second part consisted of various kinds of active enuresis (nocturnal enuresis, diurnal enuresis), primary and secondary enuresis, history of active enuresis in other children, history of urinary tract infection and respiratory infection in the child, pinworms, anal itching, seizures, diabetes, hyperactivity, breast feeding, and history of previous treatments for nocturnal enuresis. Primary nocturnal enuresis was considered as bedwetting in a child who had never been dried and secondary nocturnal enuresis was considered as experiencing dryness for more than 6 months. The data was analyzed using the SPSS 16 software. The chi-square test was applied to investigate the relationship between nocturnal enuresis and the variables, and the logistic regression was utilized to model the factors affecting nocturnal enuresis concurrently considering the significance level of 0.05.

## 3. Results

Out of a total of 710 samples, 355 were male and 355 were female. Moreover, 57 (8%) of the subjects had nocturnal enuresis, and the highest prevalence rate of nocturnal enuresis was found in the 8-year-old age group (11.9%) and the lowest rate in the 10-year-old age group (5.8%). However, no significant relationship was found between the prevalence of nocturnal enuresis and age group (*P* > 0.05). No significant difference was found regarding the decrease in nocturnal enuresis up to the age of 10 (*P* > 0.05) (Table 1). However, the difference between the prevalence rates of nocturnal enuresis in the 5- and 10-year-old age groups was significant (*P* < 0.05). In relation to gender, the prevalence of nocturnal enuresis was 10.7% in the boys and 5.4% in the girls, showing a clearly higher rate in the boys (*P* = 0.009) (Table 1).

Moreover, there were significant differences between the two genders in terms of the frequency of nocturnal enuresis and diurnal enuresis. Eleven boys (3.1%), compared with 3 girls (0.8%), had diurnal enuresis (*P* = 0.031). The frequency (intensity) of diurnal enuresis in the male children was more than that in the female ones, but no significant difference was found (*P* = 0.074). In addition, concerning the relationship between the frequency of nocturnal enuresis and age, the frequency of nocturnal enuresis in the 5-year-old age group was the highest (5.9%) (*P* = 0.061) (Table 2). Additionally, 5.2% and 2.8% of the children with nocturnal enuresis suffered from primary and secondary enuresis, respectively (based on being dry for 6 months). Moreover, 12 students (10.2%) from the preschool children and 45 students (7.6%) from the primary school children had nocturnal enuresis, showing no significant difference between educational level and nocturnal enuresis (*P* > 0.05) (Table 3). However, the frequency (severity) of nocturnal enuresis in the preschool educational level was significantly higher than that in the primary school educational level (*P* < 0.05).

Concerning the familial history of nocturnal enuresis in parents, no significant difference was found between the children with nocturnal enuresis and those without nocturnal enuresis (*P* > 0.05), while the history of nocturnal enuresis in siblings was significantly associated with nocturnal enuresis in children (*P* = 0.023). This relationship was significant for the prevalence of nocturnal enuresis as well (*P* = 0.03).

Finally, the prevalence of nocturnal enuresis in the children having one sibling (10.5%) was more than that in the children having two or more siblings (6.8%) (*P* = 0.07). Moreover, the prevalence of nocturnal enuresis was higher in the children with maternal education of high school diploma or lower (*P* = 0.01).

The results of the chi-square test showed that a number of 105 children (14.8%) of the samples had a respiratory infection, out of whom 14 children (13.3%) were enuretic, while 605 (85.2%) of the samples had no history of respiratory infection, out of whom 43 cases (7.1%) were enuretic (*P* = 0.03).

The numbers of the subjects with a history of urinary tract infection and seizures were 53 (7.5%) and 25 (3.5%) children, respectively, out of whom 5 (9.4%) and 5 (20%) children, respectively, had nocturnal enuresis as well. However, 52 (7.6%) of the children without a history of seizures and 52 (7.9%) of those with no history of urinary tract infection had nocturnal enuresis (*P* > 0.05).

Thirteen children (1.8%) had congenital problems (back problems, kidney problems, nervous problems, etc.), out of whom 2 children (15.4%) had nocturnal enuresis, while 55 children (7.9%) with no congenital problems suffered from nocturnal enuresis (*P* > 0.05).

According to the reports provided by the parents, 181 of the samples (25.5%) had deep sleep, out of whom 23 samples (12.7%) were enuretic (*P* = 0.007), while 34 children (6.4%) children with no deep sleep were enuretic.

Moreover, 78 children had been punished at school, 11 of whom (14.1%) had nocturnal enuresis (*P* = 0.036), while 46 children (7.3%) of the children who had not been punished at school had nocturnal enuresis.

The investigation of the factors associated with nocturnal enuresis showed no significant relationships between nocturnal enuresis and factors including divorce, history of nocturnal enuresis in one parent or both parents, shared bedroom, breast feeding, child's academic failure, new baby's birth, change in living place, child' age, child's birth order, educational level, maternal age, paternal age, family size, parental death, number of siblings, parental occupation, paternal education, parental kinship, history of pinworm infection, and child's hyperactivity (*P* > 0.05). However, significant relationships were found between nocturnal enuresis and factors including gender, history of nocturnal enuresis in siblings, deep sleep, respiratory infection, seizures, anal itching, and maternal education (*P* < 0.05) (Table 4). Moreover, concerning the relationship between the frequency (intensity) of nocturnal enuresis and the variables discussed, significant relationships were found only for gender, shared bedroom, deep sleep, punishment at school, history of respiratory infections, seizures, anal itching, and dominant right-handedness (*P* < 0.05). Also, diurnal enuresis was significantly associated with deep sleep, overnight nightmare, seizures, and right-handedness in the children (*P* < 0.05). The results of the logistic regression applied to evaluate the factors associated with nocturnal enuresis showed a predictive power of 92.1% for the test in identifying the factors. The results of the applied model in the logistic regression showed that male gender (*P* = 0.013, OR = 2.159, CI: 3.950–1.180), anal itching (*P* = 0.000, OR = 7.041, CI: 20.42–2.427), and seizures (*P* = 0.034, OR = 3.239, CI: 9.576–1.095) were the predictors of nocturnal enuresis in the children. Maternal education, number of siblings, history of nocturnal enuresis in siblings, deep sleep, history of pinworm infection, and respiratory infection were associated with nocturnal enuresis in the children, as detailed in Table 4.

## 4. Discussion

Nocturnal enuresis is a common developmental problem among school-aged children [5]. This study considered the DSM-IV as the criterion of nocturnal enuresis. The prevalence of nocturnal enuresis in this study was 8%. The studies conducted in Iran reported the prevalence of nocturnal enuresis in various regions in Iran to be 8.25%, 8.8%, respectively [3, 12], indicating the fact that there is no significant difference between the prevalence rates of nocturnal enuresis inside the country. However, compared with the rates in the studies conducted in Hong Kong, China, and Thailand [5, 13, 14], the prevalence rate in Iran is higher, but it is lower compared with the studies conducted in Saudi Arabia, Turkey, and Burkina Faso [15–17]. These differences could be attributed to the differences in sample size, sampling method, age range, and definition of nocturnal enuresis based on the DSM-IV or the ICD 10 (internal classification of diseases. 10) criteria. Studies conducted based on the ICD 10 criterion have reported higher rates [15, 17]. In this study, the prevalence of diurnal enuresis was 2.8%, being consistent with the study conducted by Safarinejad in 2007 [18]. In addition, a study by Toktamis et al. reported a 4.2% prevalence rate for diurnal enuresis [19].

The prevalence of diurnal enuresis in this study was significantly higher in the boys than in the girls, which is consistent with the findings of some studies [1, 19], while other studies have reported the rate of this disorder to be higher in girls than in boys [20, 21]. In the present study, the prevalence of nocturnal enuresis decreased with age with no significant relationship, whereas the difference between the prevalence rates at the ages of 5 and 10 was statistically significant. The prevalence of enuresis in this study was 10.2% at the age of 5 and 5.8% at the age of 10. This finding is compatible with the results of various studies [2, 22, 23]. Özkan et al. reported the prevalence of nocturnal enuresis in the age group of 5-6 years old to be 10.3% and at the age of 11 to be 5.6% [2]. Many studies have reported lower prevalence rates for nocturnal enuresis with age [2, 19, 24], and this difference can be attributed to different selection of age groups, being 5–10 in our study, 5–12, and occasionally 5–16, in several studies [17, 18, 25]. The prevalence of nocturnal enuresis in the boys in our study was 2.15 times higher than that in the girls, being similar with the results obtained from various studies [26–28]. However, some studies, including in China and Thailand, have not reported major differences in this regard [13, 25]. Some studies, including those conducted in Turkey and Sanandaj, have reported higher prevalence of nocturnal enuresis in girls than in boys [3, 16]. In terms of history of nocturnal enuresis in parents, no significant relationship was found between the enuretic and nonenuretic children, while there was a significant relationship between history of nocturnal enuresis in siblings and nocturnal enuresis in the children, confirming the results of other studies regarding the existence of familial history of nocturnal enuresis in enuretic children [15, 29, 30]. The results of a study conducted in Turkey by Gümüş et al. showed positive familial history of nocturnal enuresis in 75% of enuretic children [29]. The relationship between parental education and nocturnal enuresis has been discussed in some references [29, 31]. Several studies have reported lower paternal educational level and higher maternal educational level to be associated with higher prevalence of nocturnal enuresis [31]. In our study, the prevalence of nocturnal enuresis was lower in the children whose mothers had higher educational levels, whereas in Safarinejad's study the prevalence was higher in the children of the mothers with higher educational levels [18]. Other studies have also indicated the relationship between lower parental education and the prevalence of nocturnal enuresis [29]. In the present study, no significant relationship was found between paternal educational level and frequency (intensity) of enuresis, which is in accord with the result by Gunes et al.'s study in Turkey [22]. In this study, 15.7% of the children had bedwetting every night, 15.7% 4–6 times a week, 19.2% 1–3 times a week, and 49.1% less than once a week. In Gunes et al., Ozden et al., and Qing et al.'s studies, the prevalence rates for every-night bedwetting were 31%, 33%, and 24.6%, respectively [22, 32, 33]. Concerning the treatment of nocturnal enuresis in this study, 17.45% of the children consumed medicinal plants, 31.57% used chemical treatments, 24.65% used behavioral treatments including limiting fluid intake, and 26.3% expected age increase and spontaneous resolution of nocturnal enuresis. In Ramírez-Backhaus's et al.'s study, 17% of the parents did not have any treatments for nocturnal enuresis in their children, and 20% used drug treatments [26]. In the study conducted in Tehran by Safarinejad, 78.6% of the parents applied drug treatments, and this difference can be attributed to cultural differences and more parental sensitivity to the treatment of nocturnal enuresis [18]. The rates of applying treatments in Australia and New Zealand were 4.7% and 28%, respectively [21, 34]. One of the variables discussed in the relevant references is the relationship between paternal occupation and nocturnal enuresis. In our study, no significant relationship was found in this regard, being consistent with the result in Gunes et al.'s study [22]. Rona et al. in their study conducted in Scotland and England concluded that primary enuresis in first children was lower than that in subsequent children [35]. Gunes et al. did not report a significant relationship between birth order and the prevalence of primary enuresis [22]. In this study, the prevalence of nocturnal enuresis in the second children was 2.3 times higher than the rate in the only children. Being an only child seems to be a protective factor [14, 26], while it has been introduced as a risk factor in studies conducted in the US [36]. In our study, the history of urinary tract infection in the children based on parental reports was 9.4%, whereas it was reported to be 3.8% in Safarinejad's study [18], which is much lower than the rate in our study. Some studies have mentioned the ureterovesical reflux due to the contraction of the proximal ureter and the pelvic floor muscles as the cause of urinary tract infection and its relationship with higher prevalence of enuresis [22, 27, 32]. However, there was no relationship between urinary tract infection and enuresis in our study. One of the problems in children with enuresis is deep sleep and difficulty of waking up during the night [37]. This is one of the most important factors associated with the prevalence of enuresis [28]. The findings of our study also indicated a significant relationship between deep sleep and nocturnal enuresis. The results of many studies show that nocturnal enuresis is a developmental problem, while parental dealing with this problem may not be realistic [2, 15, 22, 28]. Some mothers believe that their children are able to control urination during sleep and therefore may punish them. In Özkan et al. and Safarinejad's studies, 12.8% and 26% of the enuretic children had been punished [2, 18]. In our study, 9.2% of the children with nocturnal enuresis had been punished by their parents and 14.1% by school authorities, and there was a significant relationship between corporal punishment at school and nocturnal enuresis. However, the relationship between academic failure and nocturnal enuresis was not significant although Özkan et al. reported a significant relationship between academic failure and nocturnal enuresis [2].

The application of punishment can have adverse outcomes for a child, as the study by Özkan et al. reported that the prevalence of enuresis in the children who had been trained in urination and threatened by their parents was 2.24 times higher than the rate in the children who had received encouragement [2]. In Norgaard et al.'s study, the prevalence of enuresis in the children with inappropriate parental dealing with the problem was 1.74 times higher. Therefore, inappropriate training in urination can be a risk factor of enuresis [38]. In Tai et al.'s study, a relationship was found between enuresis and overnight nightmare, being inconsistent with the results of the present study [28]. In our study, 16.7% of the enuretic children had anal itching, and the rate of nocturnal enuresis in these children was 7.04 times higher. Ghotbi and Kheirabadi in their study reported a significant relationship between anal itching and nocturnal enuresis, which may suggest an association between enuresis and oxyuriasis [3]. It has been, of course, proved that oxyuriasis is not associated with enuresis, and this may be due to the recall bias [18]. In the present study, similar to Özkan et al.'s study, no significant relationship was found between nocturnal enuresis and right-handedness; however, the intensity of nocturnal enuresis was higher in the right-handed children [2]. The prevalence of nocturnal enuresis was relatively low in this study, and this is consistent with other studies conducted in Iran. Cultural and ethnic differences may not be involved in the prevalence enuresis. In addition, the prevalence of nocturnal enuresis and some of its associated factors in our study are different from those in the studies conducted in other countries. Further studies with larger sample sizes, homogenous methods, and similar definitions of enuresis are needed to investigate the differences between the results.

## 5. Conclusion

The results of this study clearly indicated a higher prevalence rate of nocturnal enuresis in the boys than in the girls. Moreover, the frequency of nocturnal enuresis in preschool level was higher than that in primary school level. Also, history of nocturnal enuresis in siblings, deep sleep, and punishment of children at school were identified as the risk factors for nocturnal enuresis. Therefore, taking therapeutic measures and training parents in dealing with children are essential to control nocturnal enuresis.

## Figures and Tables

**Table 1:** Prevalence of nocturnal enuresis in primary school children in Khorramabad in terms of age and gender.

Variables	Nocturnal enuresis	Total	*P* value
	Number (%)	Number (%)	
Age			
5	12 (10.2)	118 (100)	0.435
6	7 (5.9)	118 (100)	
7	8 (6.8)	118 (100)	
8	14 (11.9)	118 (100)	
9	9 (7.6)	118 (100)	
10	7 (5.8)	120 (100)	
Gender			
Male	38 (10.7)	355 (100)	0.009
Female	19 (5.4)	355 (100)	

**Table 2:** Frequency of nocturnal enuresis in the children in terms of age group.

Variable	Frequency (intensity) of nocturnal enuresis					
		4 times or more a week	1–3 times a week	1-2 times a month	No nocturnal enuresis	Total
		Number (%)	Number (%)	Number (%)	Number (%)	Number (%)
Age	5	7	3	2	106	118
		5.9%	2.5%	1.7%	89.8%	100.0%
	6	3	2	2	111	118
		2.5%	1.7%	1.7%	94.1%	100.0%
	7	0	3	5	110	118
		0.0%	2.5%	4.2%	93.2%	100.0%
	8	5	1	8	104	118
		4.2%	8%	6.8%	88.1%	100.0%
	9	0	1	8	109	118
		0.0%	0.8%	6.8%	92.4%	100.0%
	10	3	1	3	113	120
		2.5%	0.8%	2.5%	94.2%	100.0%
	Total	**18**	**11**	**28**	**653**	**710**
		**2.5%**	**1.5%**	**3.9%**	**92.0%**	**100.0%**

**Table 3:** Relationship between nocturnal enuresis and educational level.

Variables	Nocturnal enuresis	Total	*P* value
	Number (%)	Number (%)	
Educational level			
Preschool	12 (10.2)	118 (100)	0.349
Primary school	45 (7.6)	592 (100)	

**Table 4:** Factors associated with nocturnal enuresis based on the logistic regression.

Variables	Enuretic	Nonenuretic	Total	*P* value	OR	95% CI
	Number (%)	Number (%)	Number (%)			
Gender						
Male	38 (10.7)	317 (89.3)	355 (100)	0.013	2.159	3.950–1.180
Female	19 (5.4)	336 (94.6)	355 (100)		—	—
Number of siblings						
0 (only child)	9 (1.5)	168 (94.9)	177 (100)	0.051	—	—
1	33 (10.5)	280 (89.4)	313 (100)		2.337	5.185–1.053
2 and more	15 (2.27)	205 (93.1)	220 (100)		1.262	3.065–0.520
Maternal education						
High school diploma and lower	48 (8.9)	486 (91.1)	534 (100)	0.018	564/2	5.609–1.172
Higher than high school diploma	9 (5.2)	167 (94.8)	176		—	—
Enuresis in siblings						
Yes	13 (14)	80 (86)	93 (100)	0.015	2.392	4.836–1.183
No	44 (7.1)	573 (92.9)	617 (100)		—	—
Deep sleep						
Yes	23 (12.7)	158 (87.3)	181 (100)	0.023	1.99	3.599–1.100
No	34 (6.4)	495 (93.6)	529 (100)		—	—
Respiratory infection						
Yes	14 (13.3)	91 (86.7)	105 (100)	0.013	2.381	4.734–1.198
No	43 (7.1)	562 (92.9)	605 (100)		—	—
Seizures						
Yes	5 (20)	20 (80)	25 (100)	0.034	3.239	9.576–1.095
No	52 (7.6)	633 (92.4)	685 (100)		—	—
Pinworm infection						
Yes	4 (5.6)	67 (94.4)	71 (100)	0.053	0.296	1.014–0.086
No	53 (8.3)	586 (91.7)	639 (100)		—	—
Anal itching						
Yes	7 (16.7)	35 (83.3)	42 (100)	<0.001	7.041	20.42–2.427
No	50 (7.5)	618 (92.5)	668 (100)		—	—
